# A grid-based map for the Biogeographical Regions of Europe

**DOI:** 10.3897/BDJ.8.e53720

**Published:** 2020-06-19

**Authors:** Marco Cervellini*, Piero Zannini*, Michele Di Musciano, Simone Fattorini, Borja Jiménez-Alfaro, Duccio Rocchini, Richard Field, Ole R. Vetaas, Severin D.H. Irl, Carl Beierkuhnlein, Samuel Hoffmann, Jan-Christopher Fischer, Laura Casella, Pierangela Angelini, Piero Genovesi, Juri Nascimbene, Alessandro Chiarucci

**Affiliations:** 1 Department of Biological, Geological and Environmental Sciences (BiGeA), University of Bologna, Bologna, Italy Department of Biological, Geological and Environmental Sciences (BiGeA), University of Bologna Bologna Italy; 2 Department of Life, Health & Environmental Science, University of L’Aquila, Coppito, L’Aquila, Italy Department of Life, Health & Environmental Science, University of L’Aquila Coppito, L’Aquila Italy; 3 Research Unit of Biodiversity (CSIC/UO/PA), Univ. of Oviedo, Mieres, Principado de Asturias, Spain Research Unit of Biodiversity (CSIC/UO/PA), Univ. of Oviedo Mieres, Principado de Asturias Spain; 4 Czech University of Life Sciences Prague, Faculty of Environmental Sciences, Department of Applied Geoinformatics and Spatial Planning, Praha, Czech Republic Czech University of Life Sciences Prague, Faculty of Environmental Sciences, Department of Applied Geoinformatics and Spatial Planning Praha Czech Republic; 5 University of Nottingham, Nottingham, United Kingdom University of Nottingham Nottingham United Kingdom; 6 Department of Geography, University of Bergen, Bergen, Norway Department of Geography, University of Bergen Bergen Norway; 7 Biogeography and Biodiversity Lab, Institute of Physical Geography, Goethe-University, Frankfurt, Germany Biogeography and Biodiversity Lab, Institute of Physical Geography, Goethe-University Frankfurt Germany; 8 Biogeography Department, University of Bayreuth, Bayreuth, Germany Biogeography Department, University of Bayreuth Bayreuth Germany; 9 School of Earth Sciences, University of Bristol, Bristol, United Kingdom School of Earth Sciences, University of Bristol Bristol United Kingdom; 10 Institute for Environmental Protection and Research, Rome, Italy Institute for Environmental Protection and Research Rome Italy

**Keywords:** biogeography, biogeographical boundaries, biodiversity monitoring, habitat, redefining, regionalisation.

## Abstract

**Background:**

Biogeographical units are widely adopted in ecological research and nature conservation management, even though biogeographical regionalisation is still under scientific debate. The European Environment Agency provided an official map of the European Biogeographical Regions (EBRs), which contains the official boundaries used in the Habitats and Birds Directives. However, these boundaries bisect cells in the official EU 10 km × 10 km grid used for many purposes, including reporting species and habitat data, meaning that 6881 cells overlap two or more regions. Therefore, superimposing the EBRs vector map over the grid creates ambiguities in associating some cells with European Biogeographical Regions.

**New information:**

To provide an operational tool to unambiguously define the boundaries of the eleven European Biogeographical Regions, we provide a specifically developed raster map of Grid-Based European Biogeographical Regions (GB-EBRs). In this new map, the borders of the EBRs are reshaped to coherently match the standard European 10 km × 10 km grid imposed for reporting tasks by Article 17 of the Habitats Directive and used for many other datasets. We assign each cell to the EBR with the largest area within the cell.

## Introduction

**Statement of the problem**

The study of the distributional patterns of biological diversity has long been a cornerstone in biogeography. These patterns are observed at many scales, from genes to ecosystems and are driven by ecological conditions, historical processes, geographic constraints and/or evolutionary processes (Lomolino et al. 2005). Dividing the geographical space into relatively-homogeneous units is one of the most traditional approaches in biogeography (Fattorini 2016). Unlike biomes, which are based on similar physiognomic vegetation structure across continents (Mucina 2019), biogeographical regions are recognised on the basis of distinct biotas, especially in terms of endemic and/or spatially clearly limited taxa and communities (e.g. Rueda et al. 2010,Morrone 2015, Fattorini 2017).

Biogeographical regions are an example of broad-scale biogeographical units; the hierarchical system in which biogeography categorises geographical areas, based on their biotas, is defined as ‘biogeographical regionalization’ (Morrone 2018). However, detecting boundaries between different biogeographical realms, regions or units is not straightforward and some recent tests using biodiversity data question the validity of traditionally-established regionalisations (Bailey 2005, Kreft and Jetz 2010,Holt et al. 2013a, Ficetola et al. 2017, Morrone 2018, Chiarucci et al. 2019). Furthermore, nature typically lacks clear discontinuities and transition zones can be recognised between adjacent biogeographical units (Walter and Breckle 2002,Holt et al. 2013b, Kreft and Jetz 2013, Ferro and Morrone 2014).

Despite such concerns, ‘biogeographical regionalisation’ is needed in order to provide reference areas for large-scale ecological analyses, as well as conservation and management practices. A prominent example is the classification of European Biogeographical Regions (EBRs), which has been adopted as an official tool for the EU Habitats Directive (92/43/EEC 1992) and Birds Directive (79/409/EEC 1979). A map of the EBRs is available on the website of the European Environment Agency (EEA 2019). This contains the official delineations used in the Habitats Directive and its extension through the EMERALD Network under the Convention on the Conservation of European Wildlife and Natural Habitats (Bern Convention).

The EBRs were delineated largely based on a map of European vegetation (Noirfalse 1987). The geographic units of that map were reclassified and vegetation types allocated to an initial set of six EBRs (Evans 2005, Evans 2012). As new Member States joined the EU, five new EBRs were then successively added. Unfortunately, a digital map of natural European vegetation did not exist when the EBRs map was created and the paper edition of *The Map of* ’*Natural Vegetation of Europe*’ was adopted (Bohn 1993, Bohn et al. 2000). Boundaries of mapping units were digitised by hand, resulting in cartographic errors (Roekaerts 2002, ETC/BD 2006). Several improved versions of the EBRs map were later produced, discussed and approved by the national experts of the Member States, resulting in the current version of the “Indicative Map of the Biogeographical Regions” adopted by the Habitats Committee in its meeting of April 2005 (ETC/BD 2006).

Although the EBRs map is now a fundamental tool for biodiversity conservation planning in Europe and is widely used by biogeographers, ecologists and conservation biologists (e.g. see Chytrý et al. 2009, Ibáñez et al. 2013, Templ et al. 2017, Ordynets et al. 2018, Chiarucci et al. 2019, Hoffmann et al. 2019), it is important to recognise that other systems have been introduced. Examples include the phytogeographical map proposed by Walter (1954) and adapted to the fauna by Freitag (1962) and the subdivision proposed by Illies (1967), Illies (1978) for the limnofauna, which remains the standard reference for studies dealing with European freshwater animals. Amongst the most recent proposals for a European biogeographical regionalisation is the Biogeographic Map of Europe by *Rivas-Martinez and Rivas-Saenz (2020)*. This includes fewer biogeographical regions, which are then divided into subregions, based primarily on endemics or spatially-delimited taxa and on a phytosociological legacy. Nonetheless, the EBRs map is the most commonly used and referenced scheme and the European Commission and the Council of Europe have adopted it for nature conservation purposes.

**Figure 1.**
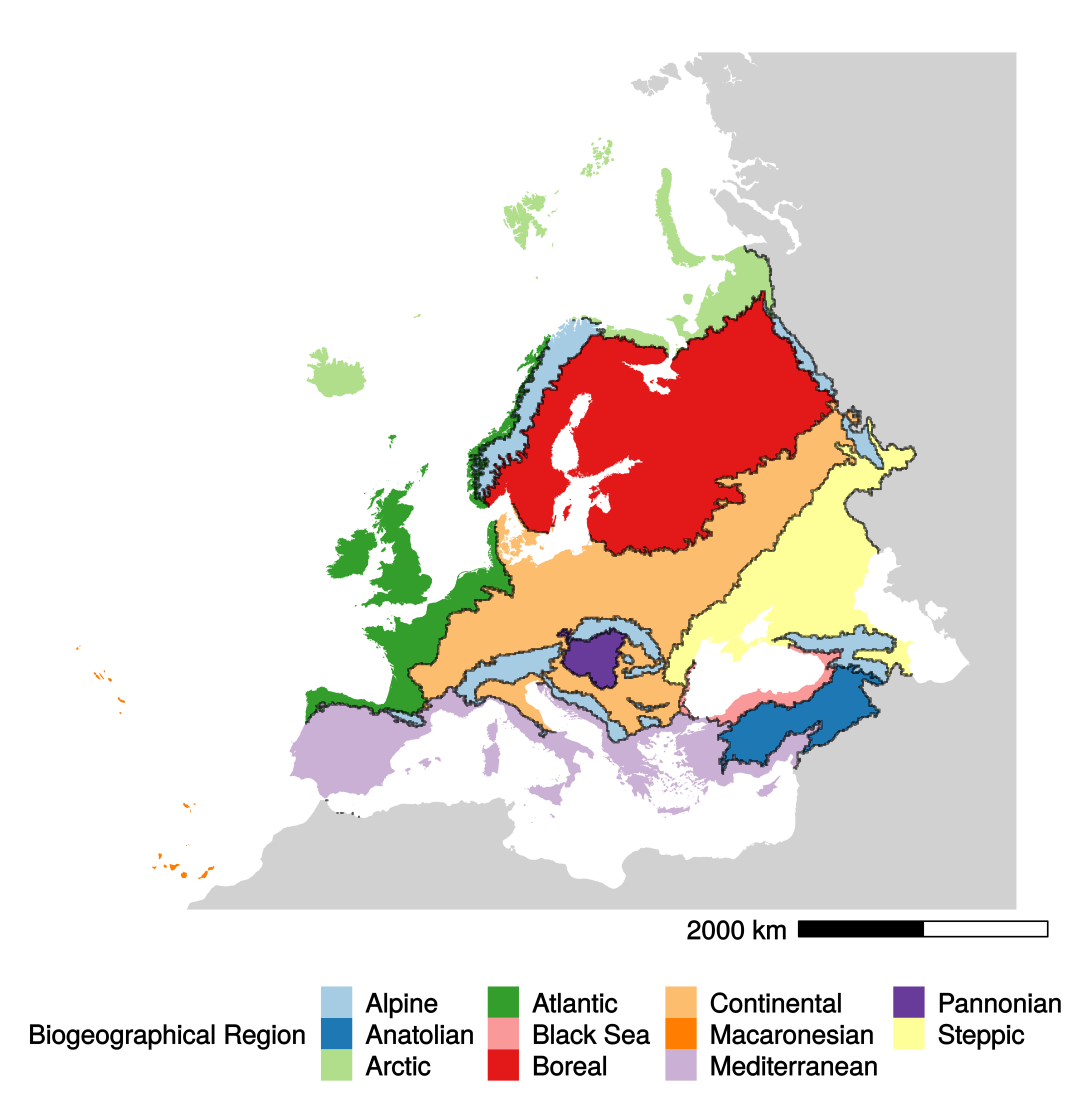
Map of the 11 European Biogeographical Regions (EBRs) showing the 7809 x 10 km × 10 km cells containing two or more EBR boundaries. These cells are displayed as empty quadrats with black borders, but given the scale, appear as black lines unless zoomed in. (The ‘outside data coverage’ is here added in grey to show the geographical boundaries with the EBRs).

The current vector format version, published on the EEA website, contains 12 EBRs, including the ‘outside data coverage’ type (EEA 2019). Operational problems arise, however, when it is used in combination with the data used for reporting species and habitat information according to Article 17 of the Habitats Directive (Eionet Portal 2019a). These data are based on a grid of 7809 x 10 km × 10 km cells that covers the European territory (EEA 2017, Eionet Portal 2019b). Indeed, superimposing the vector format EBRs map on the European grid does not allow users to unambiguously associate a substantial number (6881, ~5.87%) of the total of 117,177 individual 10 km × 10 km grid cells to corresponding EBRs, since those cells overlap two or more biogeographical regions (Fig. 1). In addition, 928 (~0.84%) cells overlap one biogeographical region plus part of the ‘outside’ data area (Fig. 1).

## General description

### Purpose

Here we present a new grid-based version of the European Biogeographical Regions map (GB-EBRs map). The dataset underpinning the GB-EBRs map is provided in Suppl. material 1. We have designed the GB-EBRs map to provide an operational tool for using the EBRs map along with grid-based data collected and used within the framework of the EU Habitats and Birds Directives. Our map aligns the EBR boundaries with the grid-cell boundaries, so that data associated with all the cells in the 10 km × 10 km grid adopted for data reporting by Article 17 of the Habitats Directive can be unambiguously assigned to the 12 EBR classes (11 EBRs plus the ‘outside data coverage’ class). Our resulting map and the related dataset are available as an open-access resource for anyone wishing to use the EBRs map and data in the context of grid-based data.

### Additional information

Our operational map represents a step towards standardising the regionalisation process of the EBRs, in compliance with the actions needed for monitoring the conservation status of habitats and species. These include the National Reports on the measures implemented and their effectiveness (Articles 11 and 17 of the Habitats Directive and Article 12 of the Birds Directive). Indeed, the monitoring of habitat types and species is strictly linked to the assessment of conservation status at the continental scale, i.e. the biogeographical regions (Ellwanger et al. 2018).

Applications of the GB-EBRs map go well beyond the data mandated by the Habitats and Birds Directives, since the same 10 km × 10 km grid is widely used for both biotic and abiotic environmental variables. Many studies use these datasets for a broad range of purposes, including spatial investigations of species and habitat distributions (e.g. Hoffmann et al. 2018), improved climatologies for environmental and ecological studies (e.g. Karger et al. 2017), biodiversity conservation and prevention of habitat loss (Maiorano et al. 2015). In addition, the GB-EBRs map could be used to answer novel research questions, such as analysing beta diversity patterns amongst and within biogeographical regions (e.g. *Chiarucci et al. 2019*).

**Figure 2.**
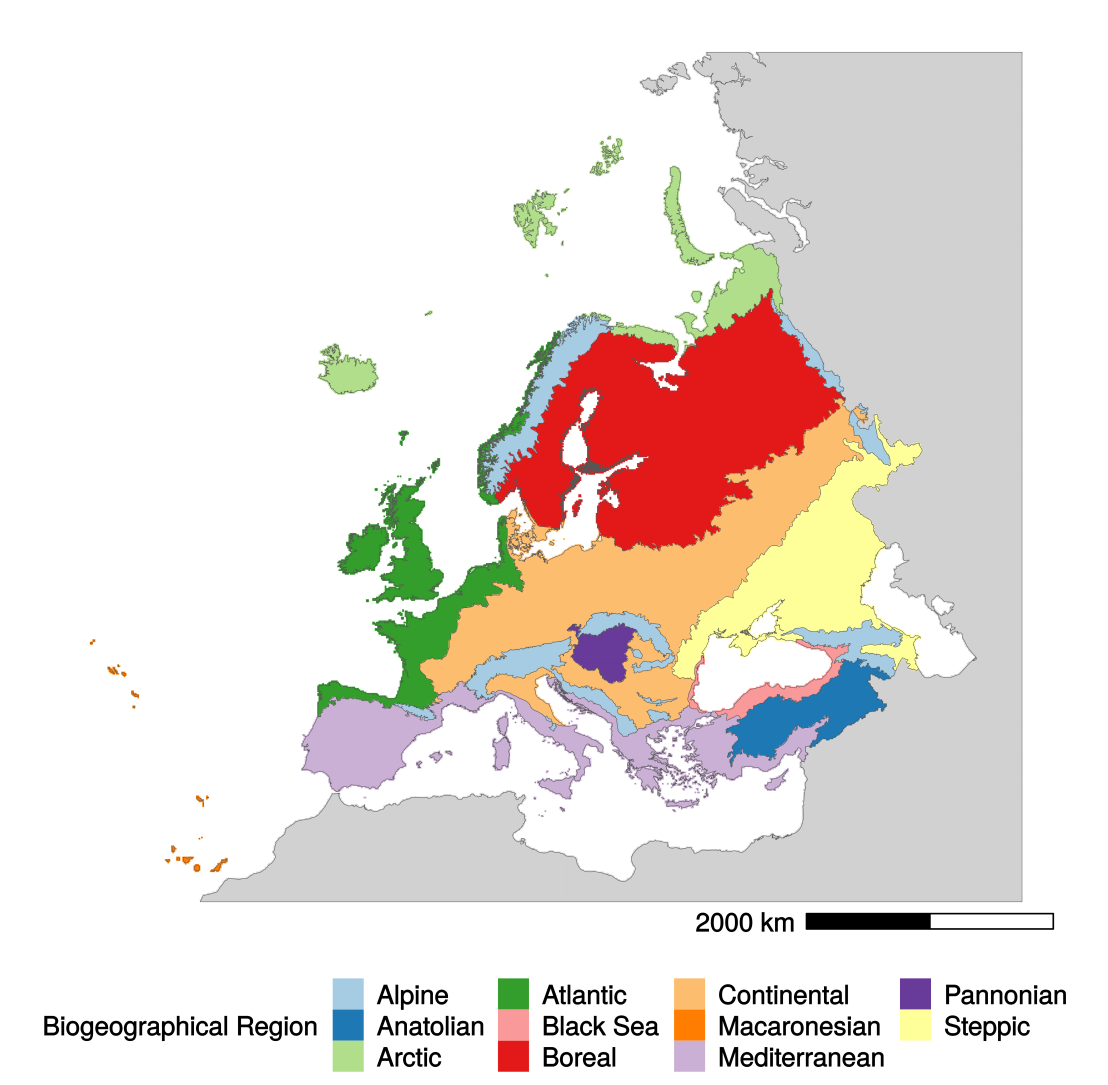
Grid-Based Map of the 11 European Biogeographical Regions (plus the ‘outside data coverage’ in grey) at 10 km × 10 km resolution (GB-EBR Map). This figure is provided only for the reader’s convenience. The raster file is available at the URL: https://zenodo.org/record/3760925#.XqBOKcgzZPY

**Figure 3.**
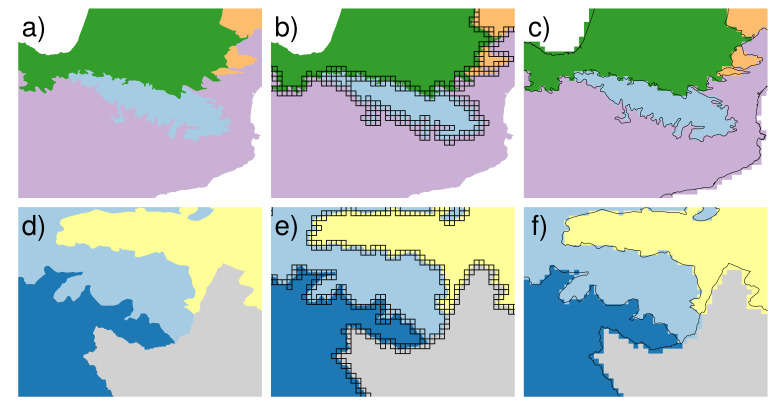
Conversion of the EBR boundaries from the EBRs (vector) to the GB-EBRs (raster) map in two different geographical zones. Top row: Pyrenees - (a) Vector boundaries of Alpine, Atlantic, Continental and Mediterranean biogeographical regions, (b) 10 km × 10 km cells that overlap two or more biogeographical regions, (c) comparison between the vector boundaries and the new grid-based ones in the GB-EBRs map. Bottom row: Caucasus - (d) Vector boundaries of Alpine, Anatolian, Steppic biogeographical regions plus ‘outside data coverage’, (e) 10 km × 10 km cells that overlap two or more biogeographical regions, (f) comparison between the vector boundaries and the new grid-based ones, reshaped in the GB-EBRs map.

The GB-EBRs map is provided in Suppl. material 2 as a raster file at 10 km resolution containing distribution and boundaries of the 12 European Biogeographical Regions (EBRs – including the ‘outside data coverage’ EBR; see Fig. 2 for an outline). Details of the differences between the EBR boundaries reported in the official version of the EBRs map and the new ones reshaped on the new raster version (GB-EBRs) are shown in Fig. 3.

The GB-EBRs map is composed of 117,177 x 10 km × 10 km cells covering the 11 EBRs (Table 1). The total area of the EBRs (sum of the surface in km^2^ for each 10 km × 10 km cell belonging to each EBR) in this GB-EBRs map is a bit larger than in the official EBRs map since the inclusion of all the land surface in the GB-EBRs map resulted in some grid cells including some ‘outside’ areas, sea and oceans. This is particularly evident for the Macaronesian region, which is the smallest one and is composed only of islands; the area of this region is 10,255 km^2^ in the official EBRs map, but the cells assigned to it in our map total 24,700 km^2^. Nevertheless, the percentage of the surface area covered by 11 regions does not statistically differ between the original EBRs and the GB-EBRs map (Wilcoxon test for paired samples, W = 61, P = 1.000).

## Sampling methods

### Sampling description

To develop the GB-EBRs map (Version 1.0), we adopted the following procedure that can be easily repeated (in case of changes in the EU territory or in the case of some eventual improvement or development of the EBRs). For each 10 km × 10 km cell in the European grid, we calculated the percentage of area covered by each EBR. Then we assigned each cell to the EBR with the largest cover value therein. Assigning each cell to a region based only on area means that the assignment is not affected by the vagaries of cell centroids (e.g. the centroid happening to be in a class covering only a small proportion of the cell), as happens with common rasterisation algorithms. We also calculated the area of each EBR both in vector (i.e. official map) and raster (i.e. new map) format, in order to investigate the size differences. Data processing was performed with R 3.6.3 (R Core Team 2019), using the packages *exactextractr* (Baston 2019), *sf* (Pebesma 2018) and *raster* (Hijmans 2019). Figures were made with *ggplot2* package (Wickham 2016) with the support of *RStoolbox* (Leutner et al. 2019), *rmapshaper* (Teucher and Russell 2020), *magrittr* (Bache and Wickham 2014) and *dplyr* (Wickham et al. 2020) packages. We finally used the package *Diplyr* (Wickham et al. 2020) to realise the table with the dataset underpinning the GB-EBR map. The R code used to produce the GB-EBR map and the related dataset is provided in Suppl. material 3

## Geographic coverage

### Description

Description: The map covers the European territory including the Azores, Madeira and the Canary Islands, but excludes seven outermost regions and the 13 overseas countries and territories.

## Usage rights

### Use license

Other

### IP rights notes

The dataset is to be used in accordance with the terms and conditions of Attribution-NonCommercial 3.0 Unported (CC BY-NC 3.0) (https://creativecommons.org/licenses/by-nc/3.0/)

## Data resources

### Data package title

Dataset of the grid-based map for the Biogeographical Regions of Europe (GB-EBR)

### Resource link

https://zenodo.org/record/3766175#.XqaHI2gzZPZ

### Alternative identifiers

https://doi.org/10.5281/zenodo.3766175

### Number of data sets

1

### Data set 1.

#### Data set name

Dataset underpinning the map entitled "A grid-based map for the Biogeographical Regions of Europe (GB-EBR)

#### Data format

Table

#### Number of columns

5

**Data set 1.** 

Column label	Column description
ID	Unique primary key
CellCode	10 km × 10 km cell identification code with reference to longitude and latitude
biogeographical_region	Biogeographical Region unambiguously associated to each 10 km × 10 km cell
X	Longitude of the 10 km × 10 km cell centroid
Y	Latitude of the 10 km × 10 km cell centroid

## Supplementary Material

C50AFB8A-280E-52A4-94CC-1BDB6863E5A2 10.3897/BDJ.8.e53720.suppl1 Supplementary material 1 Dataset of the grid-based map for the Biogeographical Regions of Europe (GB-EBR) - (Version 0.1.0) Data type dataset Brief description Here we provide the dataset underpinning the map entitled "A grid-based map for the Biogeographical Regions of Europe (GB-EBR)"
https://zenodo.org/record/3766175#.XqbP5GgzZPZ File: oo_404222.csv https://binary.pensoft.net/file/404222 Cervellini M, Zannini P, Di Musciano M, Fattorini S, Jiménez-Alfaro B, Rocchini D, Field R, Vetaas OR, Irl SDH, Beierkuhnlein C, Hoffmann S, Fischer JC, Casella L, Angelini P, Genovesi P, Nascimbene J, Chiarucci A

7BF3FD24-3CAE-5D92-9FA6-A8717213D1C2 10.3897/BDJ.8.e53720.suppl2 Supplementary material 2 A grid-based map for the Biogeographical Regions of Europe (GB-EBR) Data type raster Brief description Here we provide the grid-based map of the European Biogeographical Regions of Europe (GB-EBR). The borders of the EBRs are reshaped to coherently match the standard European 10 km × 10 km grid, thus allowing each EBR to be unambiguously assigned to a single 10 km × 10 km cell.
https://zenodo.org/record/3760925#.XqbHtGgzZPZ File: oo_404485.tif https://binary.pensoft.net/file/404485 Cervellini M, Zannini P, Di Musciano M, Fattorini S, Jiménez-Alfaro B, Rocchini D, Field R, Vetaas OR, Irl SDH, Beierkuhnlein C, Hoffmann S, Fischer JC, Casella L, Angelini P, Genovesi P, Nascimbene J, Chiarucci A

26902E94-9FA4-5D75-8CA2-9F2D6B7D3AA2 10.3897/BDJ.8.e53720.suppl3 Supplementary material 3 The R code developed to produce the GB-EBR map Data type script; text File: oo_404486.R https://binary.pensoft.net/file/404486 Zannini P

## Figures and Tables

**Table 1.** Comparison of the absolute and relative areas of each European Biogeographical Region according to the new proposed Grid-Based map (GB-EBRs Map) in comparison to the official vector map by the European Environmental Agency (EBRs Map).

	GB-BGRs Map			EBRs map	
*Biogeographical Region*	*N° of cells*	*Area* *(km²)*	*Area* *(%)*	*Area* *(km²)*	*Area* *(%)*
*Alpine*	9731	973100	8.30	948235	8.60
*Anatolian*	4468	446800	3.81	437310	3.97
*Artic*	6877	687700	5.87	568701	5.16
*Atlantic*	10127	1012700	8.64	861356	7.81
*Black Sea*	1552	155200	1.32	139682	1.27
*Boreal*	29337	2933700	25.04	2845317	25.81
*Continental*	26021	2602100	22.21	2558880	23.21
*Macaronesian*	247	24700	0.21	10255	0.09
*Mediterranean*	13694	1369400	11.69	1192947	10.82
*Pannonian*	1517	151700	1.29	151207	1.37
*Steppic*	13606	1360600	11.61	1309196	11.88
*Total*	117177	11717700	100	11023086	100
